# Gastrin-releasing peptide receptor expression in gastrointestinal stromal tumours

**DOI:** 10.1016/j.esmogo.2024.100105

**Published:** 2024-11-04

**Authors:** M. Berndsen, F. Puls, A. Thornell, Y. Arvidsson, A. Muth, S. Lindskog, E. Elias

**Affiliations:** 1 Department of Surgery, Institute of Clinical Sciences, Sahlgrenska Academy, University of Gothenburg, Gothenburg, Sweden; 2 Department of Surgery, Sahlgrenska University Hospital, Gothenburg, Sweden; 3 Department of Clinical Pathology, Sahlgrenska University Hospital, Gothenburg, Sweden; 4 Department of Laboratory Medicine, Sahlgrenska Center for Cancer Research, Institute of Biomedicine, University of Gothenburg, Gothenburg, Sweden; 5 Department of Surgery, Halland Hospital, Varberg, Sweden

**Keywords:** gastrointestinal stromal tumour, gastrin-releasing peptide, tissue microarray, peptide receptor radioligand therapy

## Abstract

**Background:**

There are limited treatment options for patients with advanced or metastatic gastrointestinal stromal tumours (GISTs) that lack mutations targetable by tyrosine kinase inhibitors (TKIs) or that have developed resistance to TKIs. Gastrin-releasing peptide receptor (GRPR) theranostics may offer a viable option in GISTs. However, the expression of the GRPR in GIST has not been extensively studied.

**Materials and methods:**

GRPR expression was evaluated using immunohistochemistry in two separate tissue microarrays from patients treated at Sahlgrenska University Hospital, one from the pre-TKI era (1983-2001) and the other from the post-TKI era (2014-2020). In total, 205 tumour samples were characterized as having low/none or moderate/high expression of the GRPR, and these were correlated with clinical characteristics and survival outcomes.

**Results:**

In total, 80% of the tumour samples exhibited moderate or high expression of GRPR. GRPR expression was not associated with gender, age, tumour location, or risk group, as defined by the modified National Institutes of Health (NIH) consensus criteria. Neoadjuvant treatment with TKI was correlated with low/none GRPR expression (*P* = 0.04). In patients who underwent surgery with curative intent and did not receive neoadjuvant treatment, GRPR expression was not associated with survival outcomes.

**Conclusions:**

This study is the first to investigate GRPR expression in a large cohort of GIST tumours. Our results demonstrate that most GIST tumours exhibit a moderate to high expression of the receptor, suggesting that GRPR theranostics could be a viable option for TKI-resistant GIST. Interestingly, tumours that were pretreated with TKI showed lower expression levels of GRPR, indicating a need for further studies to explore this finding.

## Highlights

• Advanced GISTs that are resistant to standard treatments have limited treatment options.
• We investigated the expression of GRPR in a large cohort of GIST tumours.
• Nearly 80% of the tumour samples exhibited moderate to high levels of GRPR expression.
• GRPR theranostics could thus be a viable option for advanced GIST.

## Introduction

Gastrointestinal stromal tumours (GISTs) are intestinal mesenchymal malignancies thought to arise from the interstitial cells of Cajal.^1^, ^2^, ^3^ GISTs most commonly occur in the stomach but can be found anywhere in the gastrointestinal tract. Small, low proliferative GISTs are often cured with surgery alone.^4^, ^5^, ^6^ Advanced GISTs are treated with a combination of surgery and tyrosine kinase inhibitors (TKIs) if the tumour has a susceptible mutation in the genes coding for the tyrosine kinase receptors *KIT* or *platelet-derived growth factor receptor alpha* (*PDGFRA*).^7^^,^^8^ Metastatic patients treated with TKIs are prone to acquire secondary mutations that lead to TKI resistance.^9^^,^^10^ Only limited therapeutic options are available for metastatic patients who either do not initially harbour a treatment-sensitive oncogenic mutation or develop resistance.^4^^,^^11^

The gastrin-releasing peptide (GRP) is a neuropeptide that induces gastrin secretion in the stomach and mediates gastrointestinal motility.^12^ It also causes the release of several different hormones and neurotransmitters and is linked to various homeostatic and behavioural regulations.^13^ The gastrin-releasing peptide receptor (GRPR) expression has been demonstrated in GIST and in breast and prostate cancers.^14^^,^^15^

GRPR has recently been proposed as a potential peptide receptor-mediated radiotherapy (PRRT) target. A small study demonstrated specific binding of the antagonist NeoB to the GRPR in three GIST cell lines, which was also confirmed by immunohistochemistry.^16^ Recently, several studies have evaluated GRPR-targeting molecules, particularly the antagonist ^68^Ga-NeoB, for the development of GRPR radiopeptide theranostics.^17^^,^^18^ GRPR-targeted imaging has shown promise in prostate cancer and estrogen receptor-positive breast cancer.^19^ A small study evaluating nine patients with advanced GIST showed variable uptake on ^68^Ga-NeoB-PET. The uptake was correlated with the vitality of the lesion.^20^

Based on available data, PRRT targeting GRPR could represent a novel treatment option for GIST and benefit the subgroup of TKI-resistant patients with limited treatment options. However, to our knowledge, the expression of the PRRT-target protein GRPR has not been characterized in a broader cohort of patients with GIST.

In this study, we aim to assess the expression patterns of GRPR in a large cohort of patients with GIST. We also link the GRPR expression to clinical characteristics to determine if it represents a potential novel prognostic marker.

## Material and methods

### Patient cohorts

For this study, a new post-TKI tissue microarray (TMA) was created, including tumour samples from 134 individual patients undergoing surgery for GIST at Sahlgrenska University Hospital between 2014 and 2020. As a reference cohort, a historical pre-TKI GIST TMA was used, including 256 tumour samples from 243 patients who underwent surgery at the same hospital between 1983 and 2001.^21^ This pre-TKI cohort represents patients treated before the introduction of TKI, whereas the post-TKI TMA included samples from patients treated after TKI had become standard practice in the clinical care of patients with GIST.

Clinical parameters investigated in this study were patient specific, including sex, age at diagnosis, TKI treatment, and survival status. The tumour-specific parameters were tumour location, tumour size, mitotic frequency, immunohistochemistry, and mutational status. The modified NIH consensus criteria^1^^,^^22^ were used for both cohorts and, based on our previous work, the cohorts were dichotomised into high risk or other.^6^

### Tissue microarrays

The post-TKI TMA was constructed as follows: formalin-fixed and paraffin-embedded tumour tissue was collected from the Department of Clinical Pathology and Genetics, Sahlgrenska University Hospital. Sections from the tumour blocks were placed on glass slides and stained with haematoxylin and eosin. A senior sarcoma pathologist (FP) identified the tumour material on the glass slides for guidance. The TMA was constructed with 0.6-mm diameter cores. Each core utilized the Simport M473 T-Sue Microarray Mold Kits according to the manufacturer’s instructions (Simport Scientific Inc, Saint-Mathieu-de-Beloeil, Quebec, Canada). Immunostaining for CD117/c-KIT and DOG1 was carried out to validate that the cores were representative of tumour tissue. The block included normal tissue from the stomach, small intestine, and colon as negative controls.

Clinical details regarding patient characteristics, treatment, and follow-up data were previously collected from medical journals in a database and were available for this study.^6^ The construction description of the historical pre-TKI TMA has been published previously.^21^

### Immunohistochemistry

Sections (3-4 μm) from the paraffin blocks were placed on glass slides and treated in Dako PT-Link (DakoCytomation, Glostrup, Denmark) using Dako EnVision FLEX Target Retrieval Solution (high pH). The following primary antibodies were used: GRPR recombinant rabbit monoclonal antibody (catalogue number 703928; Invitrogen (Carlsbad, CA); diluted 1:500), CD117 (c-KIT) rabbit polyclonal antibody [catalogue number A4502; Dako Agilent (DakoCytomation); diluted 1:300], and DOG1 mouse monoclonal antibody [catalogue number NCL-L-DOG-1; Triolab (Molndal, Sweden); diluted 1:100].

Immunohistochemical staining was carried out in a Dako Autostainer Link using Dako EnVision FLEX according to the manufacturer’s instructions (DakoCytomation). Dako EnVision FLEX+ (LINKER) rabbit or mouse antibodies were used for all staining.

When evaluating GRPR expression patterns it was noted that these closely resembled the previously described staining patterns of the KIT receptor.^23^ In brief, six different staining patterns were detected and assessed for each tumour.

The patterns of GRPR immunostaining were as follows: A, diffuse strong staining; B, strong staining with a dotted pattern; C, membrane staining; D, dotted pattern and weak cytoplasmic staining; E, diffuse weak staining, and F, GRPR-negative GIST.

Three of the six staining patterns (A, B, and C) exhibited a strong intensity coupled with a membranous protein expression and for further analysis, these were grouped into a single group (moderate/high). The remaining staining patterns (i.e. D, E, and F) were grouped as none/low. Assessment was carried out by two blinded observers (YA and EE).

### Statistical analysis

Univariate analysis was carried out with a chi-square test for categorical variables and a Student’s *t*-test for continuous variables. The recurrence-free survival (RFS) was defined as the time from surgery to recurrence. Patients without recurrence were censored at the latest date of follow-up or death from other causes. The time between the date of diagnosis and GIST-related death was calculated as the disease-specific survival (DSS). Patients were censored at the latest follow-up or at the date of unrelated GIST death. Overall survival (OS) was calculated from the date of diagnosis to the date of death. Survival was analysed using Kaplan–Maier curves, and a comparison between groups was carried out with the log-rank test. The association between survival and various clinical parameters, including the GRPR expression, was studied using univariate and multivariate (Cox regression) analyses. All *P* values were two-sided, with a *P* value <0.05 considered statistically significant.

All statistical analyses were carried out using R version 4.1.2 (R Foundation for Statistical Computing, Vienna, Austria) and R Studio (PositTM, PBC, Boston, MA).

The study was approved by the Swedish Ethical Review Authority (approval number 2022-06273-02).

## Results

### Clinical characteristics of the cohorts

In the pre-TKI TMA, 138 tumour samples from 128 patients were available for GRPR expression analysis. This cohort consisted of 65 females and 63 males with a mean age of 68 years (range 30-92 years). None had received neoadjuvant treatment with TKI before surgery. Of these, 114 had resections with curative intent, while 14 were palliative resections. The median follow-up was 69 months (range 0-355 months).

Among the 134 tumour samples in the post-TKI TMA, 77 were available for analysis of GRPR. The patient cohort consisted of 40 females and 37 males with a mean age of 68 (49-86) years. Of these, 21 patients (27%) had received neoadjuvant treatment with TKI (imatinib) before surgery. All but two resections were carried out with curative intent. The median follow-up was 24 months (range 0-92 months).

Table 1 presents the clinical characteristics of the two TMA cohorts. The most common tumour site was the stomach. The modified NIH consensus criteria were used to stratify the patients according to the risk of relapse. Approximately half of the patients in the pre-TKI TMA were in the high-risk group, compared with 29% in the post-TKI TMA. In both cohorts, the most common mutation was found in *KIT* exon 11. Because of its historical nature, tumours with no identifiable mutation (wild type) were over-represented in the pre-TKI TMA. Mutation analysis was not performed on three tumours in the pre-TKI TMA and six tumours in the post-TKI TMA. Among the five tumours with an imatinib-resistant mutation, all but one had moderate to high GRPR expression.

**Table 1:** Clinical characteristics of the pre- and post-TKI TMAs

Clinical characteristics	Pre-TKI TMA (*n* = 128)	Post-TKI TMA (*n* = 77)
Tumour site		
Gastric	75 (59)	50 (65)
Nongastric	53 (41)	27 (35)
Risk group (NIH)		
High	63 (49)	22 (29)
Other	65 (51)	55 (71)
Mutation		
KIT exon 11	81 (63)	43 (56)
PDGFRA	3 (2)	13 (17)
Other^a^	5 (4)	5 (7)
Wild type	36 (28)	10 (13)
Imatinib-resistant mutation	—	5 (7)
Neoadjuvant imatinib	—	21 (27)
Relapse	31 (24)	3 (4)
Noncurative resections	14 (11)	2 (3)
Death due to GIST	41 (32)	4 (5)
Median (range) follow-up (months)	69 (0-335)	24 (0-92)

Values are *n* (%) unless otherwise specified.

GIST, gastrointestinal stromal tumour; TKI, tyrosine kinase inhibitor; TMA, tissue microarray; NIH, the National Institutes of Health; PDGFRA, platelet-derived growth factor receptor alpha.

a Includes KIT exons 9, 10, and 17, and BRAF.

### GRPR expression

**Figure 1:**
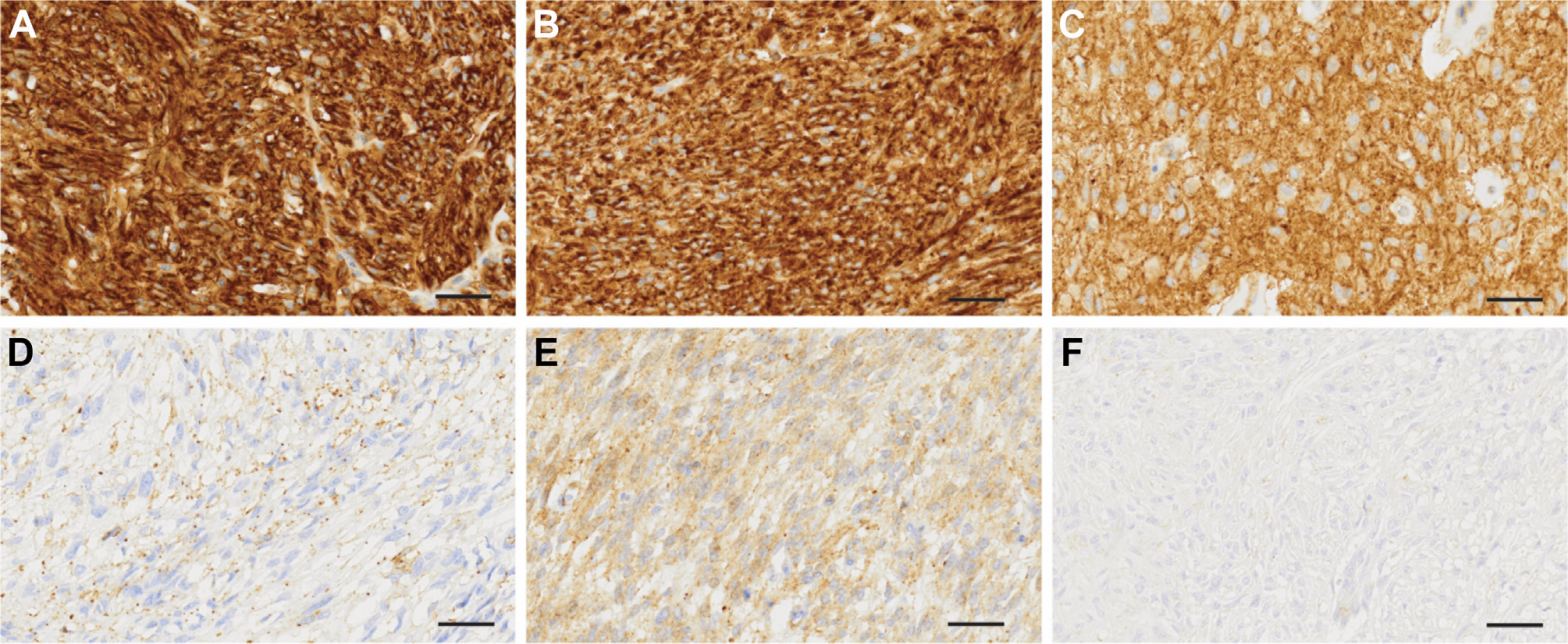
**Patterns of gastrin-releasing peptide receptor (GRPR) immunostaining in gastrointestinal stromal tumour.** (A) Diffuse strong staining. (B) Strong staining with a dotted pattern. (C) Membrane pattern. (D) Dotted pattern with weak cytoplasmic staining. (E) Diffuse week staining. (F) GRPR-negative GIST. Scale bar = 40 μM.

Figure 1 demonstrates the different immunostaining patterns: A, B, and C belong to the moderate or high expression group, whereas D, E, and F belong to the low or none expression group. The two TMA cohorts were combined to analyse the clinical characteristics according to GRPR expression (Table 2, *n* = 205). In the combined cohort, 80% of the tumours exhibited a moderate or high GRPR expression. In the univariate analysis, the GRPR expression was not correlated with the patient’s sex, age, tumour location, risk group, or mutational status. The GRPR expression was significantly lower in tumours pretreated with imatinib than those without pretreatment (*P* = 0.04).

**Table 2:** Clinical characteristics according to GRPR expression

Clinical characteristics	Low/none (*n* = 41)	Moderate/high (*n* = 164)	*P* value^a^
Sex (female:male)	20:21	85:79	0.9
Mean age (years)	70	68	0.2
Stomach:other^b^	30:11	95:69	0.11
High risk:other^c^	19:22	66:98	0.6
KIT exon 11:other^d^	22:19	97:67	0.7
Neoadjuvant imatinib (yes:no)	8:33	13:151	0.04

Values are *n* unless otherwise specified.

GRPR, gastrin-releasing peptide receptor; NIH, the National Institutes of Health; PDGFRA, platelet-derived growth factor receptor alpha.

a The chi-square test was used for categorical variables, except for neoadjuvant imatinib, for which Fisher’s exact test was applied. The Student’s *t*-test was used for age.

b Includes the oesophagus, duodenum, small bowel, colon, rectum, and extraintestinal.

c According to the modified National Institutes of Health consensus criteria, other includes intermediate, low, and very low risk.^6^

d Includes *KIT* exons 9, 10, and 17; *PDGFRA* 12 and 18; and wild type.

### Survival analysis

**Figure 2:**
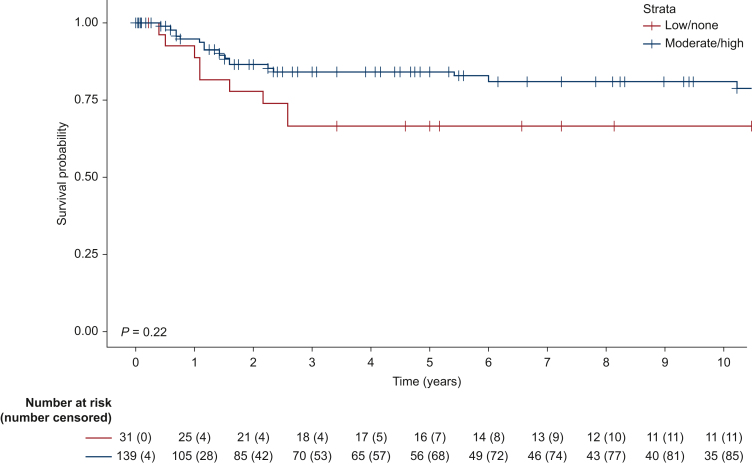
Kaplan–Meier curve of recurrence-free survival in 170 patients who underwent surgery for gastrointestinal stromal tumour without neoadjuvant tyrosine kinase inhibitor treatment, grouped by gastrin-releasing peptide receptor expression.

Survival analyses were conducted on a combined cohort of both TMAs, including patients who underwent curative intent resections without neoadjuvant imatinib treatment (*n* = 170). There was no significant difference in DSS, OS (*P* = 0.19 versus *P* = 0.71; Supplementary Figure S1A and B, available at https://doi.org/10.1016/j.esmogo.2024.100105), or RFS (*P* = 0.22; Figure 2) based on GRPR expression patterns (low/none versus moderate/high). The risk of recurrence was higher for patients in the high-risk group, with other tumour sites than the stomach, who belonged to the pre-TKI cohort or harboured a *KIT* exon 11 mutation (Table 3). However, sex, age, and GRPR expression were not statistically associated with RFS in the univariate analysis. In the multivariate analysis, the correlation between RFS and the risk group was statistically significant with a hazard ratio of 0.02 (95% confidence interval 0.002-0.12).

**Table 3:** Clinical characteristics compared between patients with and without recurrence after surgery for GIST

Clinical characteristics	No recurrence (*n* = 139)	Recurrence (*n* = 31)	Univariate analysis^a^	Multivariate analysis^b^*,* HR (95% confidence interval)	*P* value
Sex					
Male	68	17	*P* = 0.6		
Female	70	14			
Mean age (years)	68.7	67.7	*P* = 0.6		
Risk score (NIH)					
High	33	30	*P* < 0.001		
Other	106	1		0.02 (0.002-0.12)	<0.001
Site					
Stomach	92	14	*P* = 0.05		
Other	47	17		1.3 (0.6-2.8)	0.47
Mutation					
KIT exon 11	71	23	*P* = 0.04		
Other	68	8		0.8 (0.3-1.7)	0.51
GRPR					
Low/none	22	9	*P* = 0.2		
Moderate/high	117	22			
Cohort					
Pre-TKI	56	0	*P* = 0.004	−	−
Post-TKI	83	31			

Values are *n* unless otherwise specified.

GIST, gastrointestinal stromal tumour; GRPR, gastrin-releasing peptide receptor; HR, hazard ratio; NIH, the National Institutes of Health; TKI, tyrosine kinase inhibitor.

a Univariate analysis was carried out with chi-square for categorical variables and Student’s *t*-test for continuous variables (age).

b Variables that were statistically significant in the univariate analysis were included in the multivariate analysis, which was conducted using Cox regression analysis that demonstrated an HR with a 95% confidence interval and a *P* value.

The DSS was significantly lower in the high-risk group compared with other risk groups (*P* < 0.0001) and in the pre-TKI cohort compared with the post-TKI cohort (*P* = 0.014; see Supplementary Figure S2A-F, available at https://doi.org/10.1016/j.esmogo.2024.100105). Furthermore, the OS was statistically lower in the high-risk group (*P* = 0.042) and in the pre-TKI cohort (*P* = 0.009; see Supplementary Figure S3A-F, available at https://doi.org/10.1016/j.esmogo.2024.100105).

## Discussion

In our cohort of 205 patients who underwent primary surgery for GIST, 80% of the tumours expressed GRPRs with high or moderate intensity. There was no correlation between GRPR expression and the clinical characteristics of the patients or tumours. However, tumours in patients treated with imatinib expressed significantly lower levels of GRPR. RFS, OS, and DSS were similar in patients whose tumours expressed GRPR compared with those whose tumours did not. Therefore the expression of GRPR was not a prognostic marker for survival.

GRPR expression has not previously been studied in a large cohort of GIST tumours. Paulmichl et al.^16^ demonstrated a specific binding of the antagonist NeoBOMB1 to the GRPR in three GIST cell lines, which was confirmed by immunohistochemistry. GRPR expression has also been characterized in several other tumour types.^17^^,^^24^ Morgat et al.^25^ studied GRPR expression in 1432 breast cancer samples, with overexpression of the GRPR observed in 83.2% of estrogen receptor-positive breast cancers. The same group also demonstrated a positive correlation between the binding of the ^68^Ga-antagonist and GRPR expression in breast cancer.^26^ To date, there have been several promising preclinical and clinical trials evaluating GRPR-targeting imaging and therapy in prostate and breast cancers.^18^^,^^27^

GRPR-targeted theranostics is also an emerging field for GIST. However, the largest clinical trial to date, the Mitigate project, included only nine patients with advanced GIST, who were evaluated for radiotracer uptake using a GRPR antagonist radioligand, which exhibited variable uptake.^20^ Thus, although it seems likely that GISTs commonly express GRPR, additional studies are required to correlate protein expression levels with GRPR antagonist radioligand uptake. If confirmed, this could lead to a potential treatment option through peptide receptor radioligand therapy (PRRT), similar to how ^177^Lu-DOTATATE has been used in treating metastatic neuroendocrine tumours.^28^

Interestingly, GISTs treated with neoadjuvant imatinib before surgery exhibited lower levels of GRPR. This could suggest that *either* GRPR expression correlates with certain characteristics influencing the decision to offer neoadjuvant TKI treatment *or* that GRPR expression is causally affected by TKI treatment. As GRPR expression was not linked to risk grading or mutational status, it is plausible that TKI treatment itself downregulates GRPR expression. Ideally, tumour samples taken before and after TKI treatment could be used to determine this. The loss of GRPR expression suggests that potential GRPR-mediated therapy would primarily benefit patients who have not received TKI treatment. Furthermore, this finding emphasises the complex interplay between TKI treatment and molecular pathways associated with GIST progression. In the Mitigate project, the authors also speculate whether treatment regimens are linked to the variable receptor density observed in advanced GIST.^20^

By linking protein expression levels to clinical outcomes, we also assessed GRPR expression as a novel prognostic marker. When tumours were grouped according to high or low GRPR expression, there was no correlation with other known risk factors such as age, location, *KIT* exon 11 mutation, or risk score stratification. In addition, GRPR expression did not significantly predict RFS survival in our cohort. However, there was a small difference in RFS survival between groups and a type 2 error cannot be ruled out.

Regarding the possible role of GRP signalling as an oncogenic mechanism in GIST, our finding of a high prevalence of moderate/high GRPR expression in GISTs is noteworthy. Drawing from evidence in other cancers,^14^^,^^18^^,^^24^^,^^29^ this finding underscores the possibility of GRPR signalling playing a role in GIST pathogenesis, thereby potentially fuelling tumour growth and progression. However, the lack of correlation between GRPR expression and traditional clinical parameters such as gender, age, tumour location, or mutation status suggests a unique role for GRPR in GIST biology.

The strengths of this study are the large, well-characterized cohort of patients surgically treated for GIST from different treatment eras with long follow-ups and comprehensive analysis. However, several limitations should be acknowledged. The mutational analysis of the tumours in the pre-TKI TMA is biased towards wild-type tumours because of the older sequencing techniques used, which resulted in limited coverage of relevant genes. Furthermore, as the TMAs were constructed using tumour tissue from primary surgeries, GRPR expression in patients with metastatic GIST and those with acquired TKI resistance in a palliative setting was not studied. Thus, the interesting question of whether GRPR expression is affected by TKI resistance could not be addressed. In addition, the functional significance of GRPR expression in GIST pathogenesis warrants further investigation, including *in vitro* and *in vivo* studies, to elucidate its role in tumour biology and the therapeutic modulation of TKI therapy.

### Conclusions

GRPR expression is prevalent in GIST tumours, and this study supports GRPR as a potential target for peptide–receptor-based imaging and therapeutics. Our findings reveal a possible negative influence of prior TKI treatment on GRPR expression levels, suggesting that in further studies of GRPR-mediated therapy in advanced GIST, GRPR treatment should precede TKI treatment.
