# Supplementary material for: Gastrin-releasing peptide receptor expression in gastrointestinal stromal tumours
Source: ESMO Gastrointest Oncol. 2024 Nov 4;6:100105. doi: 10.1016/j.esmogo.2024.100105 (PMC12836489; doi:10.1016/j.esmogo.2024.100105)
Supplement: Supplementary data [file mmc1.docx]

Supplemental material

1A


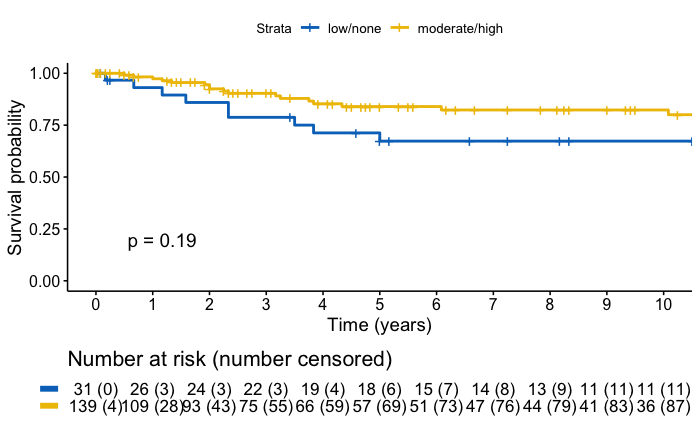


1B


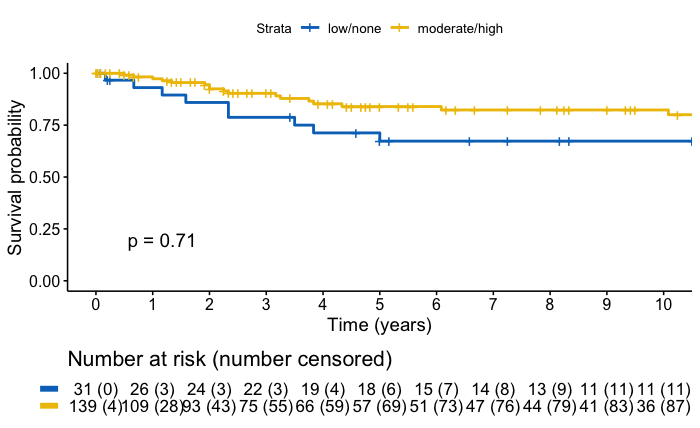


Supplemental figure 1 Kaplan-Meier curve of A) disease specific and B) overall survival in 170 patients operated for GIST without neoadjuvant TKI treatment grouped according to GRPR expression.

2A


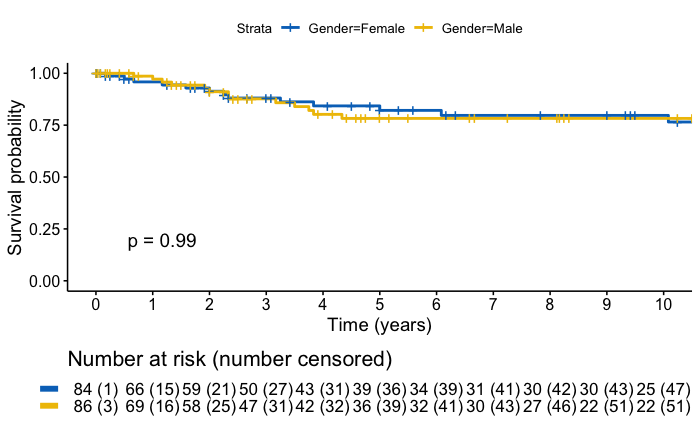


2B


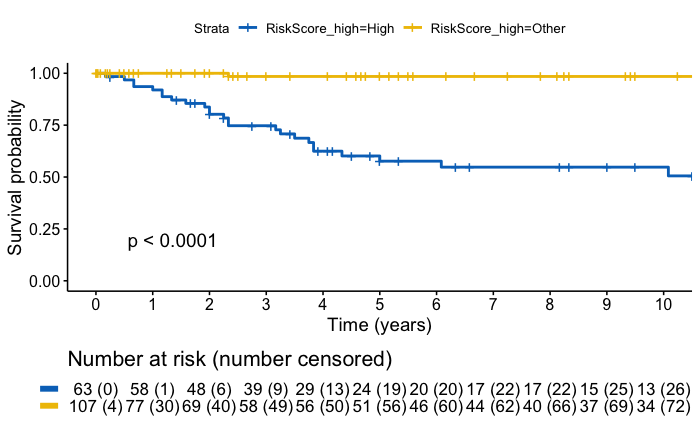


2C


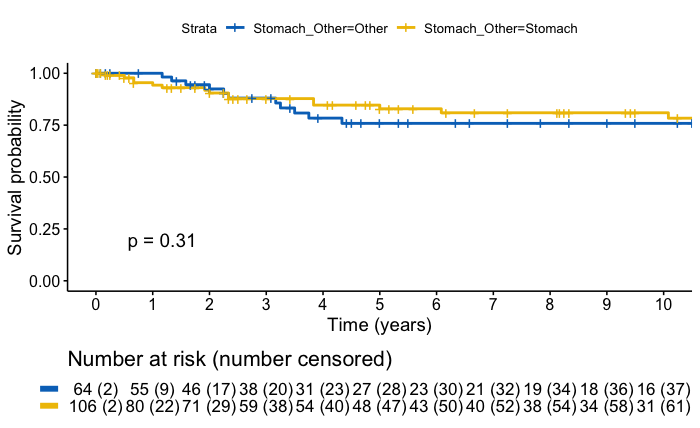


2D


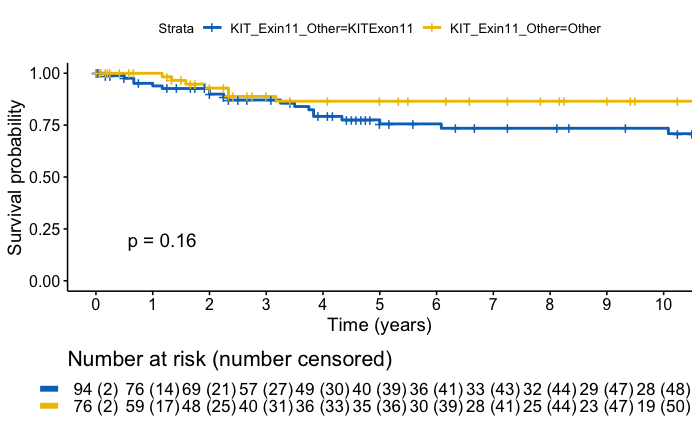


2E


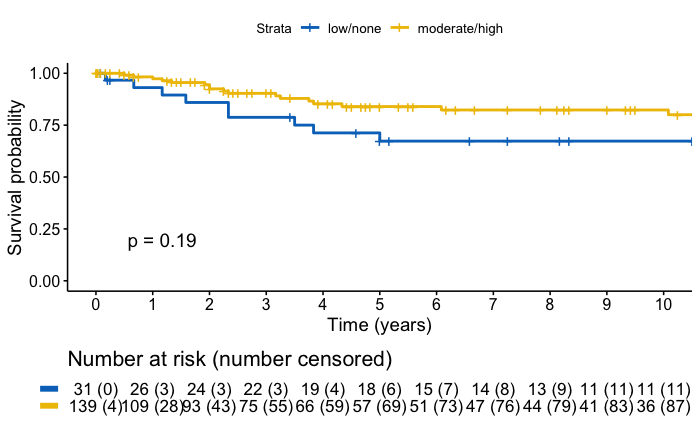


2F


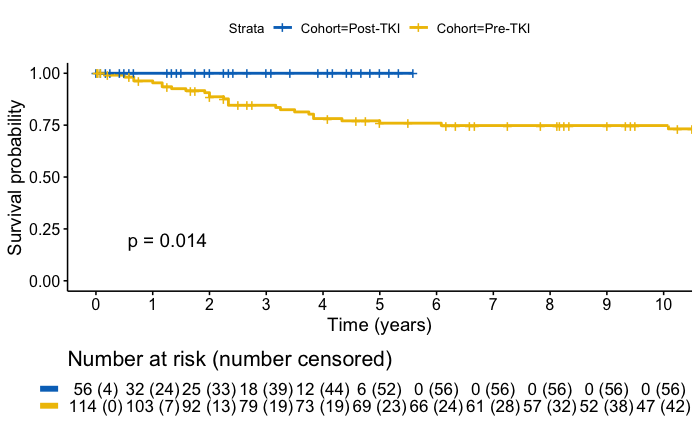


Supplementary figure 2. Kaplan-Meier curve of disease specific survival in 170 patients operated for GIST without neoadjuvant TKI treatment grouped according A) gender, B) risk score, C) tumour site, D) mutation E) GRPR expression and F) Pre vs post TKI cohort.

3A)


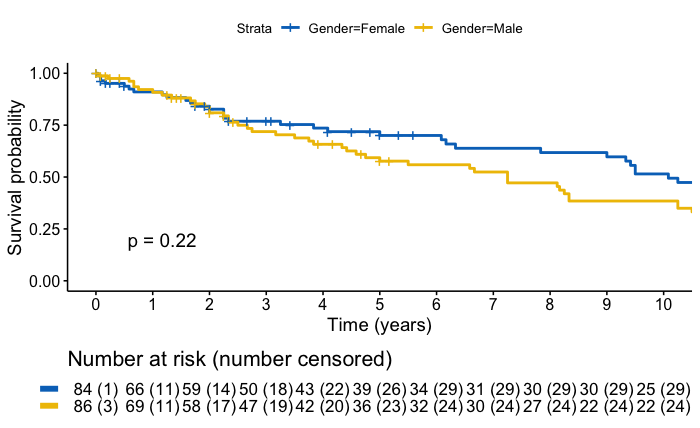


3B)


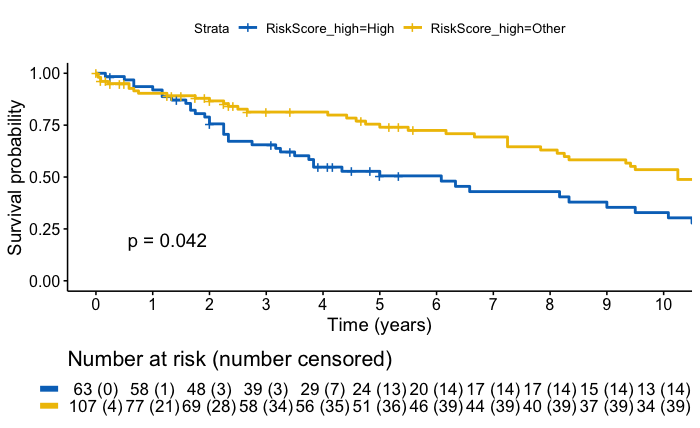


3C)


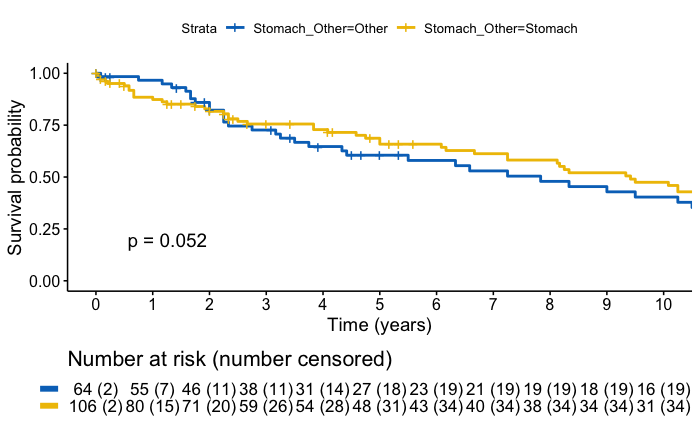


3D)


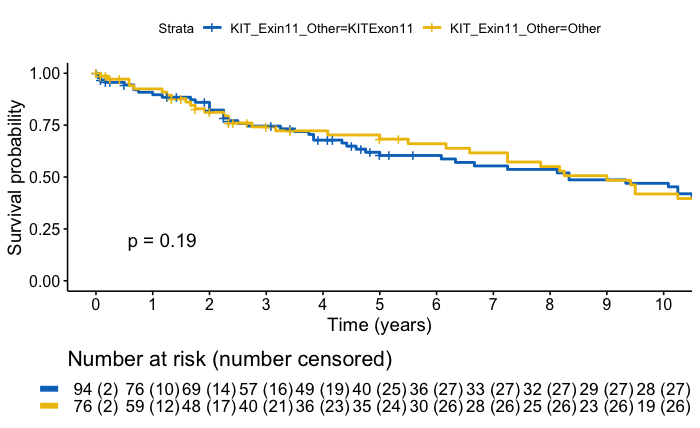


3E)


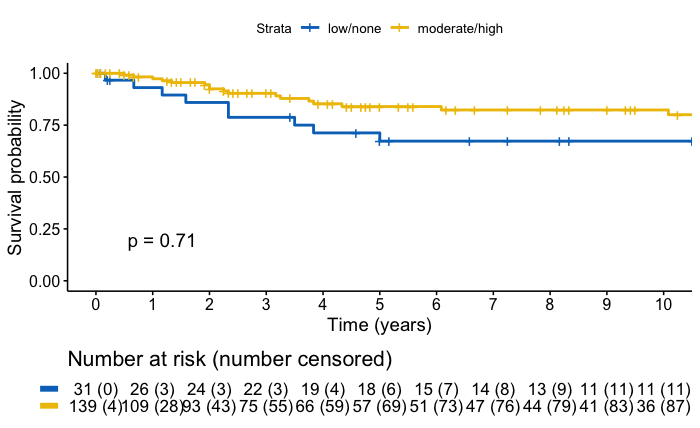


3F)


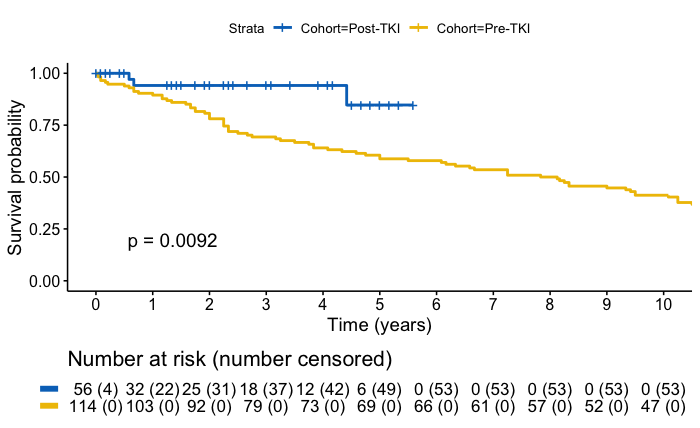


Supplementary figure 3. Kaplan-Meier curve of overal survival in 170 patients operated for GIST without neoadjuvant TKI treatment grouped according A) gender, B) risk score, C) tumour site, D) mutation E) GRPR expression and F) Pre vs post TKI cohort.
